# Supplementary material for: Experience of Older Patients with COPD Using Disease Management Apps: A Qualitative Study
Source: Healthcare (Basel). 2024 Apr 7;12(7):802. doi: 10.3390/healthcare12070802 (PMC11011793; doi:10.3390/healthcare12070802)
Supplement: Supplementary file 1 [file healthcare-12-00802-s001.zip › File S2:interview outline.pdf]

File S2：interview outline

Questions

1. How do you know about the disease management APP? What did you think of the disease management APP when you first know it?

2. Why did you choose to use/not use the disease management APP?

3. What barriers have you encountered in using the disease management APP to manage your disease process? How did you overcome these obstacles?

4. What do you think are the benefits of using the disease management APP to manage diseases? Can you tell me more about it?

5. What impact do you think the use of disease management APP has brought to your life? Why?
